# Fabrication Process of Bistable Electrowetting Displays Based on Photolithography and Inkjet Printing

**DOI:** 10.3390/mi17090990

**Published:** 2026-08-22

**Authors:** Guisong Yang, Benyou Wang, Zhiqiang Chang, Hongwei Jiang

**Affiliations:** 1 School of Electronic Information and Artificial Intelligence, West Anhui University, Lu’an 237012, China; 2 Guangdong Provincial Key Laboratory of Optical Information Materials and Technology and Institute of Electronic Paper Displays, South China Academy of Advanced Optoelectronics, South China Normal University, Guangzhou 510006, China

**Keywords:** bistable electrowetting display, non-planar electrode, inkjet printing, silver nanoparticle ink

## Abstract

Bistable electrowetting displays (BEWDs) have become a competitive technical solution for low-power and static display fields due to their unique zero-power-consumption holding characteristics. However, traditional BEWD pixels face problems such as high driving voltage, complex processes, high cost, open structures, and difficulty in high-resolution array fabrication. Aiming at these bottlenecks, this paper proposes a closed stereoscopic bistable EWD pixel structure driven by planar and non-planar electrodes, and completes the design, fabrication, and performance verification of the device. The fabrication process proposed in this paper combines photolithography and inkjet printing, featuring simple process, low cost, closed pixels, and low driving voltage, which solves the key problems of traditional processes. The research results provide a feasible process route and experimental basis for the development of low-power bistable electrowetting display devices.

## 1. Introduction

Electrowetting displays are a promising reflective display technology because of their low power consumption [1,2,3], high white state reflectance [4], fast switching [5], flexibility [6], multi-gray scale [7,8], bi-stability [9,10] and full color [11,12]. The oil/water two-phase microfluidics in pixels of BEWDs feature two stable states [10,13,14,15]. Therefore, pixel structures must be fabricated to confine the oil/water two-phase microfluidics within pixels, such that power is consumed only during the switching between on and off states. This greatly reduces the device refresh rate and the duration of applied electric fields, thereby lowering damage to the dielectric layer and hydrophobic layer of the device. However, achieving bi-stability requires a more sophisticated design of the EWD device structure, which introduces challenges to the manufacturing process. Charipar et al. [14] patterned planar BEWD electrodes E1 and E2 via laser direct writing and fabricated bistable pixel structures using single-step photolithography. Nevertheless, laser direct writing is inefficient and costly, making it unsuitable for mass production. The planar electrodes are located at the bottom of the pixel, and driving the oil/water microfluidics on the surface of a 20 μm thick photoresist requires a high driving voltage (>100 V). The pixels they fabricated are of an open structure, which cannot switch individual pixels in a pixel array independently, hindering their application in high-resolution displays such as imaging.

Han Zhang et al. [16] used vacuum thin-film deposition (magnetron sputtering, PECVD), photolithographic patterning, and wet etching to form planar aluminum electrodes at the bottom of photoresist and non-planar aluminum electrodes on the photoresist surface, respectively, and fabricated BEWD pixels. Although non-planar electrodes reduced the driving voltage, the vacuum coating equipment is expensive, the fabrication process involving multiple photolithography and etching steps is complex with high material consumption, and the fabricated pixels remain open-structured. Based on the simulated model parameters and theoretical analysis in the previous work [17], this paper fabricates a unique closed stereoscopic BEWD pixel. A two-step photolithography technique is adopted. The first photolithography forms pixel grooves, and the second photolithography constructs pixel walls, resulting in a closed stereoscopic BEWD pixel structure. A comparison is made between the performance of driving microfluidic motion of planar electrodes (E1, E2) and non-planar electrodes (E1, E2′, on the photoresist surface). The inkjet printing of silver nanoparticle ink was employed, which directly forms patterned silver thin-film electrodes E2′ after heating, greatly simplifying the fabrication process and reducing costs. Oil filling via inkjet printing enables drop-on-demand dispensing, solving the problems of uneven filling, overfilling, and underfilling in conventional EWD oil filling under water.

## 2. Fabrication Process of BEWD Pixels

**Figure 1:**
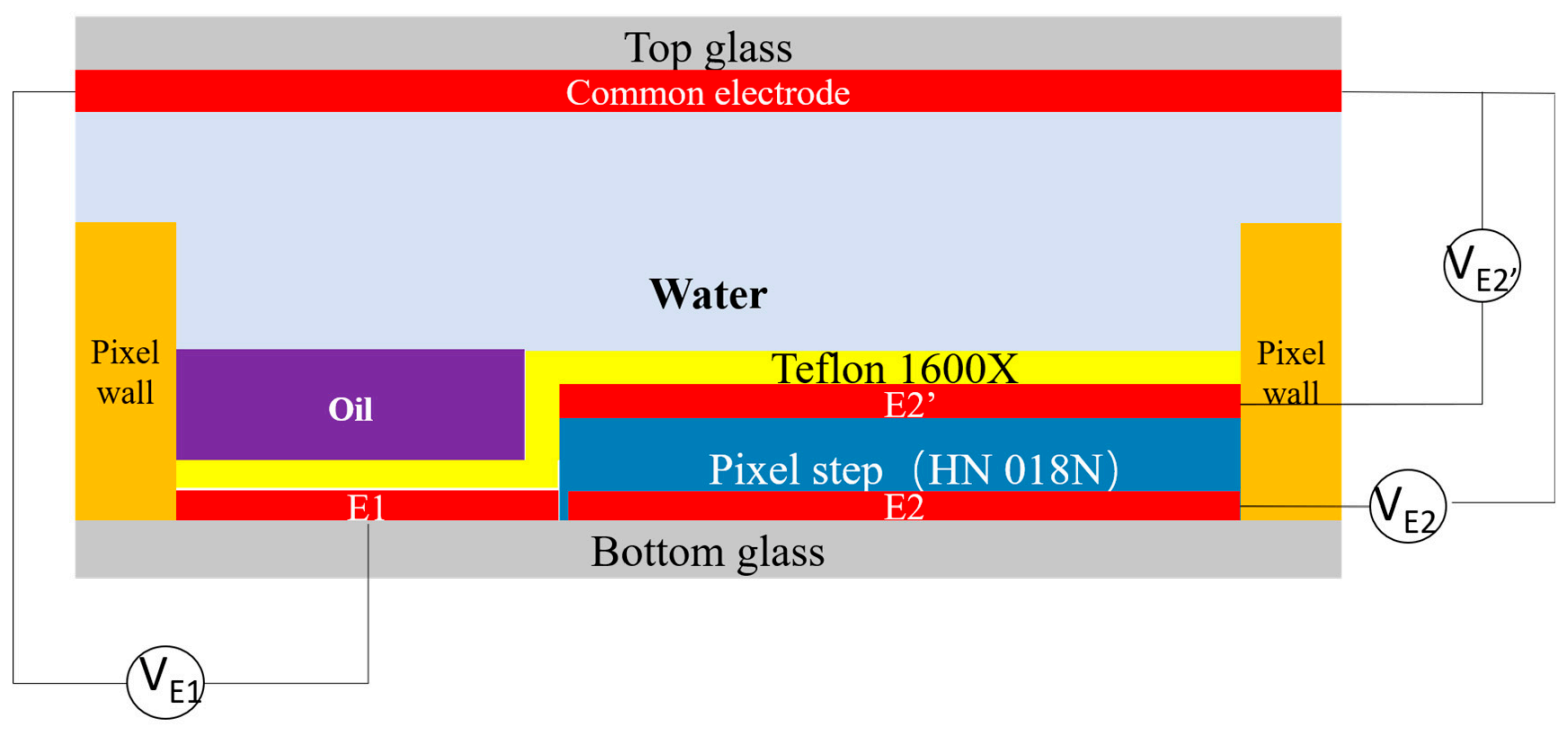
The cross-section view of the bistable electrowetting display pixel structure.

Figure 1 shows the cross-section view of the three-dimensional BEWD pixel structure with quarter-circle electrode 1 (E1) at the bottom of the pixel grooves, notched electrode 2 (E2) at the bottom of the pixel step, and notched E2′ on the top surface of the pixel step. The voltage applied on non-planar E1 and E2′ controls the oil droplet in the water phase and causes it to move between the pixel grooves and the pixel step, confined in an independent square pixel wall, showing the potential of bi-stability.

**Figure 2:**
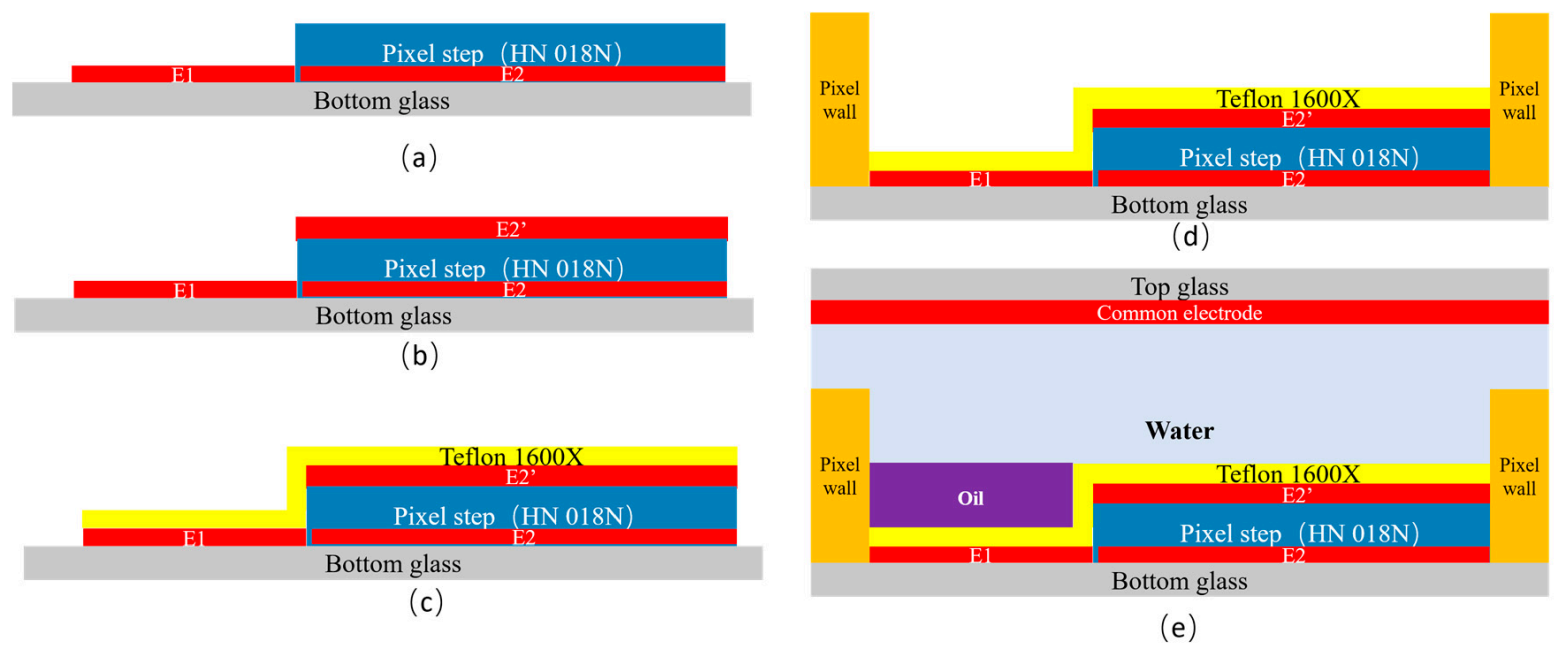
Fabrication process of bistable electrowetting display pixels. (**a**) First photolithography for stereoscopic pixel grooves. (**b**) Inkjet printing of patterned silver electrodes. (**c**) Coating of Teflon AF1600x hydrophobic layer. (**d**) Second photolithography for pixel wall construction. (**e**) Inkjet printing oil filling and packaging.

The main device fabrication process includes first photolithography for stereoscopic pixel grooves, inkjet printing of patterned silver electrodes, coating of Teflon AF1600x hydrophobic layer, second photolithography for pixel wall construction, and inkjet printing oil filling and packaging, as shown in Figure 2.

(a) First Photolithography for Stereoscopic Pixel Grooves

Photoresist exposure and development are used to fabricate bistable EWD stereoscopic pixel grooves. A negative acrylic resin photoresist HN 018N, jointly developed by South China Normal University and Suntific Microelectronic Materials Co., Ltd. (Weifang, China), is adopted. A customized pixel groove mask is used to expose and develop the photoresist to obtain pixel groove array microstructures. The photolithography process mainly includes photoresist spin coating, photoresist pre-baking, exposure, development, and thermal curing. Through experiments and analysis, the optimal process parameters were determined, and 12 μm high stereoscopic bistable pixel grooves were fabricated on the dual-electrode ITO substrate. The optimized process parameters are listed in Table 1.

(b) Inkjet Printing of Patterned Silver Electrodes

The patterned silver electrodes need to be deposited on the upper surface of the 12 μm thick photoresist HN 018N with pixel groove arrays. Since the HN 018N photoresist is weakly hydrophilic (contact angle > 80°), hydrophilic treatment is required to ensure better spreading of inkjet-printed silver ink droplets on the photoresist surface to form uniform films. The common hydrophilic treatment for HN 018N photoresist in the laboratory is UV–Ozone treatment, which increases oxygen-containing groups and surface roughness, reducing the contact angle. The UV–Ozone treatment time is 10 min. Due to the instability of wettability caused by water and air on the photoresist surface after UV–Ozone treatment (i.e., hydrophilic modification has a certain timeliness), the substrate treated with UV–Ozone should be immediately subjected to patterned silver electrode inkjet printing on the photoresist surface.

In this paper, the DMP 2850 piezoelectric inkjet printer is used for depositing patterned electrode E2′ on the photoresist surface. Commercial silver nanoparticle ink DGP 40TE 15C, supplied by Sigma Aldrich (Shanghai, China), is adopted. It consists of silver nanoparticles, alcohol-based solvent, and dispersant, with a silver content of 30–35 wt.% (weight percent), silver nanoparticle size of 30–50 nm (nanometer), viscosity of 10–18 cps (centipoise), and surface tension of 35–38 dynes/cm (dyne per centimeter). Through experimental optimization, successful inkjet printing of Ag nanoparticle ink on the photoresist surface was achieved, and thermal curing (200 °C, 10 min) formed a uniformly thick metallic silver film electrode E2′ array.

(c) Coating of Teflon AF1600x Hydrophobic Layer

The hydrophobic insulating layer is coated on the bistable electrowetting display substrate with stereoscopic pixel groove arrays to provide a hydrophobic surface for the device, enabling water droplets to form a large initial contact angle. This allows wide range regulation of water droplet wettability upon electric field application, which significantly contributes to the switching performance, driving voltage, reliability, and other technical parameters of electrowetting display devices.

In this study, spin coating is used to form a single-layer DuPont’s Teflon AF1600x hydrophobic insulating film of approximately 0.8 μm thickness for bistable electrowetting display pixels. A Smart coater10 numerical control spinner is used for coating at 1420 rpm for 65 s. The coated substrate is pre-heated on a hotplate at 85 °C for 3 min to evaporate most solvents, and then placed in an oven at 185 °C for 30 min to remove all solvents, forming a Teflon AF1600x film of approximately 800 nm thickness with a hydrophobic surface. After fabricating the ITO planar electrodes E1 and E2 and the non-planar E2′ silver electrode on the 12 μm high photoresist surface via inkjet printing on the bistable electrowetting display glass substrate, Teflon AF1600x is spin-coated on the entire substrate to provide a large initial water contact angle and act as an insulating dielectric layer for driving oil/water two-phase microfluidic motion.

(d) Second Photolithography for Pixel Wall Construction

The pixel walls based on hydrophilic photoresist HN 018N are constructed on the hydrophobic Teflon AF1600x surface of the stereoscopic bistable electrowetting display pixels to form a closed stereoscopic bistable EWD pixel array. Since the previously coated Teflon AF1600x fluoropolymer film is hydrophobic, it has poor adhesion to the hydrophilic pixel wall material HN 018N. Direct coating of weakly hydrophilic photoresist on the Teflon AF1600x surface results in weak adhesion. To enhance adhesion between the photoresist pixel walls and the hydrophobic layer, hydrophilic modification of the hydrophobic Teflon AF1600x fluoropolymer surface is required to convert it from hydrophobic to hydrophilic. Dry etching was employed to modify wettability, increasing oxygen containing hydrophilic groups and surface roughness of the hydrophobic fluoropolymer. The spin-coating speed and time of HN 018N are 1000 rpm and 65 s, respectively, and exposure time is 10 s.

After pixel wall construction via photolithography, the hydrophilic surface of Teflon AF1600x was restored to its original hydrophobic state via high-temperature reflow.

(e) Inkjet Printing Oil Filling and Packaging

This step completes quantitative inkjet printing filling of purple oil into the fan-shaped grooves of the stereoscopic bistable electrowetting display pixel array and final device packaging. The volume of the fan-shaped groove is calculated from the designed device dimensions. Each ink droplet has a volume of 10 pL, which allows us to determine the required number of inkjet droplets for each three-dimensional pixel groove. The DMP 2850 inkjet printer is loaded with self-developed purple ink, and stable ink droplet ejection is achieved by adjusting the piezoelectric driving waveform. After inkjet printing filling, the substrate is placed in a 15 °C low-temperature environment to freeze and fix the ink in the pixel grooves, and then polar conductive liquid is filled on the oil surface to complete oil/water two-phase microfluidic filling in the bistable EWD pixels.

Finally, the upper cover plate is attached to the fabricated bistable stereoscopic pixel lower substrate, and the oil and water are firmly encapsulated between the upper and lower substrates with encapsulant. This completes the fabrication of the electrowetting display device with bistable stereoscopic pixels. The device is left at room temperature for approximately 6 h to allow the frozen oil to return to ambient temperature before power-on testing.

## 3. Results and Discussion

### 3.1. Effect of UV–Ozone on Photoresist Surface Wettability

**Figure 3:**
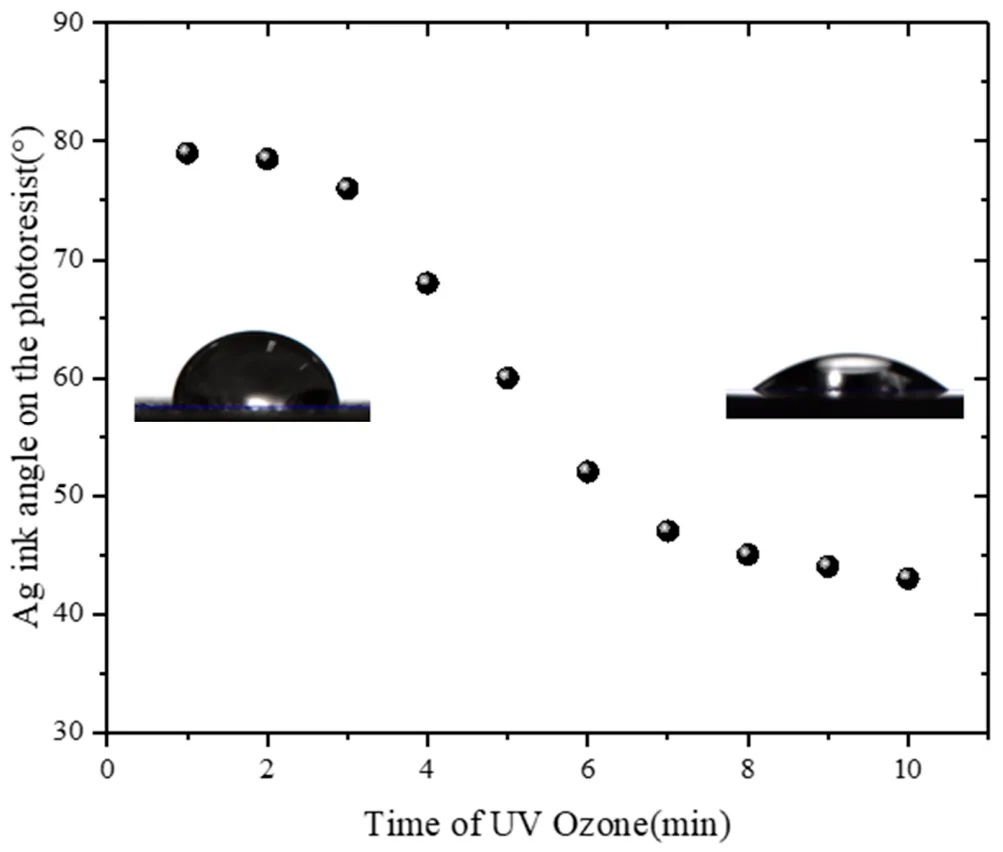
Effect of UV–Ozone treatment time on contact angle of silver nanoparticle ink on HN-018N photoresist surface.

The 5 μL silver nanoparticle ink droplets are pipetted onto substrates treated with different UV–Ozone times, and contact angle changes are observed and recorded using a POWER EACH contact angle measuring instrument. As shown in Figure 3, the contact angle of the HN-018N photoresist surface decreases significantly with increasing UV–Ozone treatment time, gradually changing from weakly hydrophilic to highly hydrophilic.

The contact angle decreases slowly from 0 to 3 min (from 79.5° to ~75°), drops sharply from 3 to 7 min (from ~75° to ~45°), and then decreases gradually. After 10 min of UV–Ozone treatment, the contact angle stabilizes at approximately 41°, meeting the requirements for good spreading of silver ink to form a uniform E2′ silver electrode wet film, which is thermally cured into a silver electrode pattern.

### 3.2. Effect of Voltage on Inkjet-Printed Silver Line Width

We analyze the four stages of inkjet formation driven by piezoelectric waveforms, which critically affect the jetting morphology of silver nanoparticle ink. With fixed Slew Rate (slope, V/μs) and Pulse Duration T (μs), the effect of amplitude (voltage variation in Phase 2) on the line width of dried silver films formed by inkjet-printed silver ink droplets is investigated.

**Figure 4:**
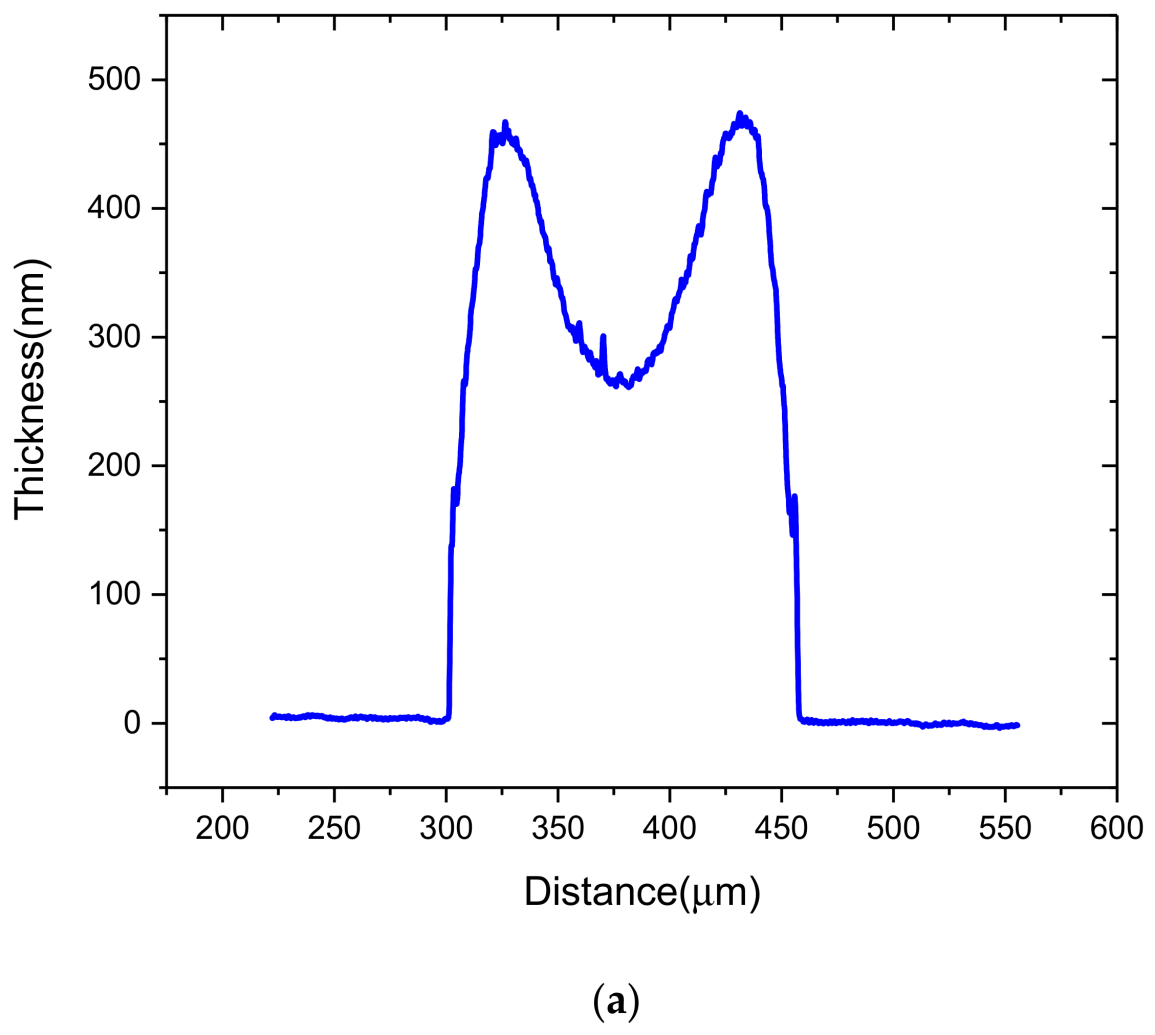

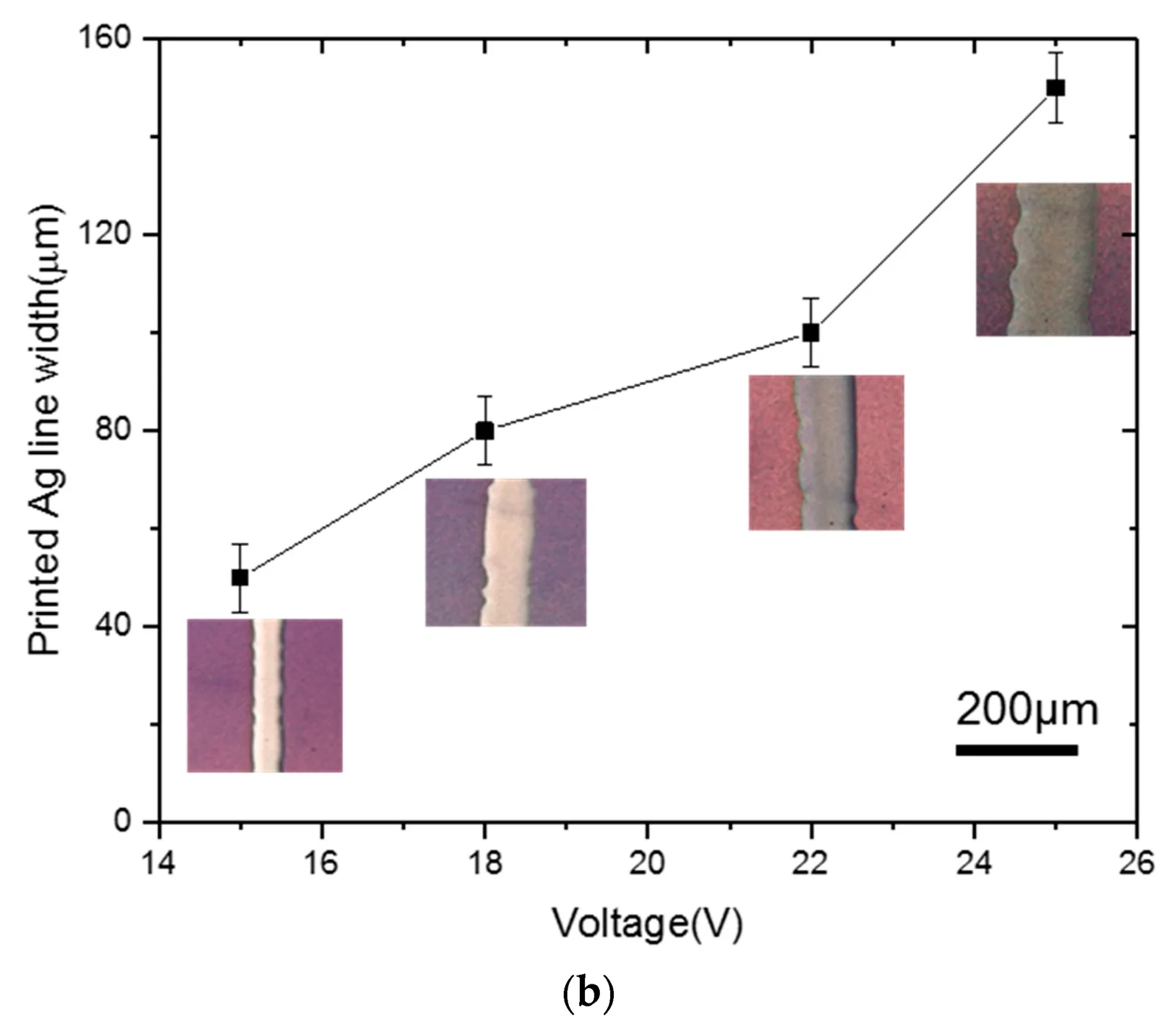
Characterization of inkjet-printed silver nanoparticle film line width. (**a**) Thickness and width measurement data of silver lines. (**b**) Line width of silver nanoparticle ink printed at different driving voltages.

A Dektak XT (BRUKER, Billerica, MA, USA) stylus profilometer is used to measure the thickness and width of printed silver lines. At a printing voltage of 25 V (volt), the average thickness of the silver film is ~400 nm, and the average width is ~150 μm (Figure 4a). With Slew Rate fixed at 0.5 V/μs and T at 15 μs, the line width of silver lines deposited on the HN-018N photoresist surface is compared as printing voltage varies from 15 V to 25 V (Figure 4b).

The silver line width increases with piezoelectric driving voltage, from 50 μm (15 V) to 150 μm (25 V). These data provide guidance for fabricating conductive silver electrodes via inkjet printing of silver nanoparticle ink. For high-precision (high-resolution) electrode patterns, reduce the driving voltage to form fine, discrete droplets that connect into lines and films. For rapid large-area silver ink film deposition on HN-018N photoresist, appropriately increase the driving voltage.

### 3.3. SEM and EDS Characterization

**Figure 5:**
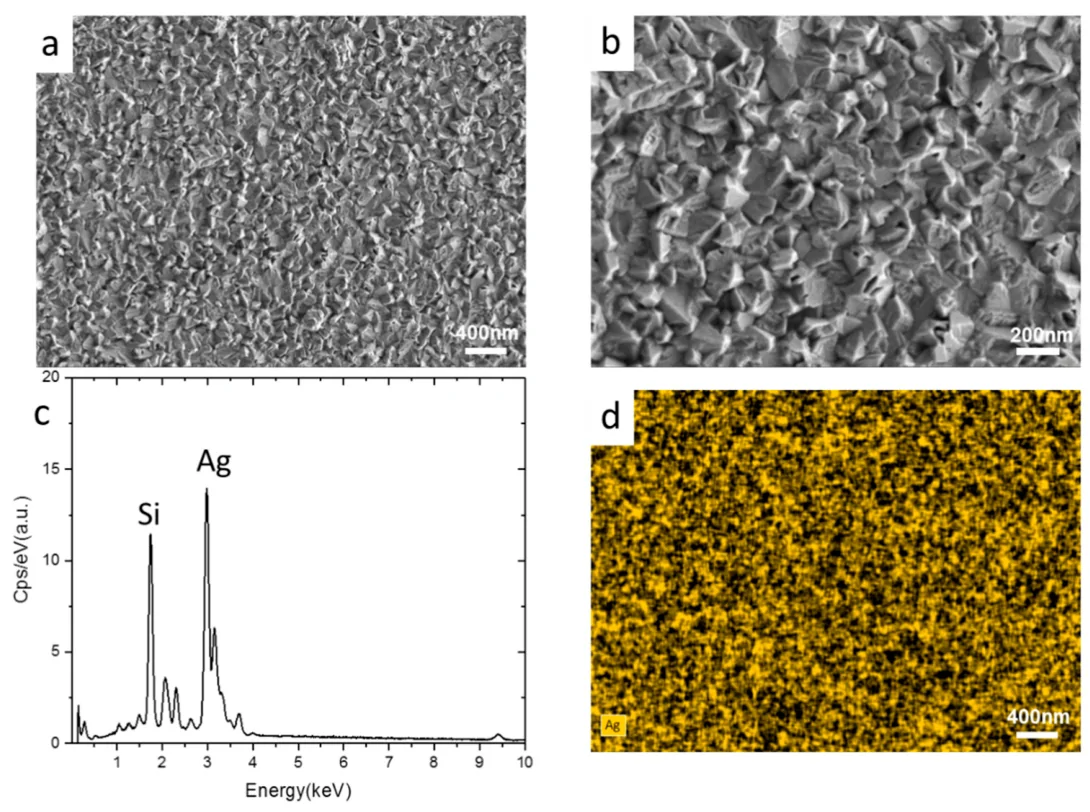
SEM and EDS characterization results of silver nanoparticle films. (**a**) SEM image (×20,000 magnification). (**b**) SEM image (×50,000 magnification). (**c**) EDS spectrum. (**d**) Ag elemental mapping.

To further investigate the microscopic surface morphology and composition of inkjet-printed silver nanoparticle films, SEM is used to observe cured silver nanoparticle films, and EDS is used to analyze their composition, as shown in Figure 5.

Figure 5a,b show SEM images of cured silver nanoparticle films at 20,000× and 50,000× magnification, respectively. Cured silver nanoparticles (~50 nm) are tightly packed randomly after organic solvent evaporation at high temperature, forming a conductive silver film. EDS analysis (Figure 5c) shows the film mainly contains silver (Ag) and silicon (Si), with trace carbon (C), oxygen (O), aluminum (Al), and other impurities and residual organics. Figure 5d is the Ag elemental mapping from EDS scanning.

### 3.4. Effect of Curing Temperature on Sheet Resistance of Silver Ink

**Figure 6:**
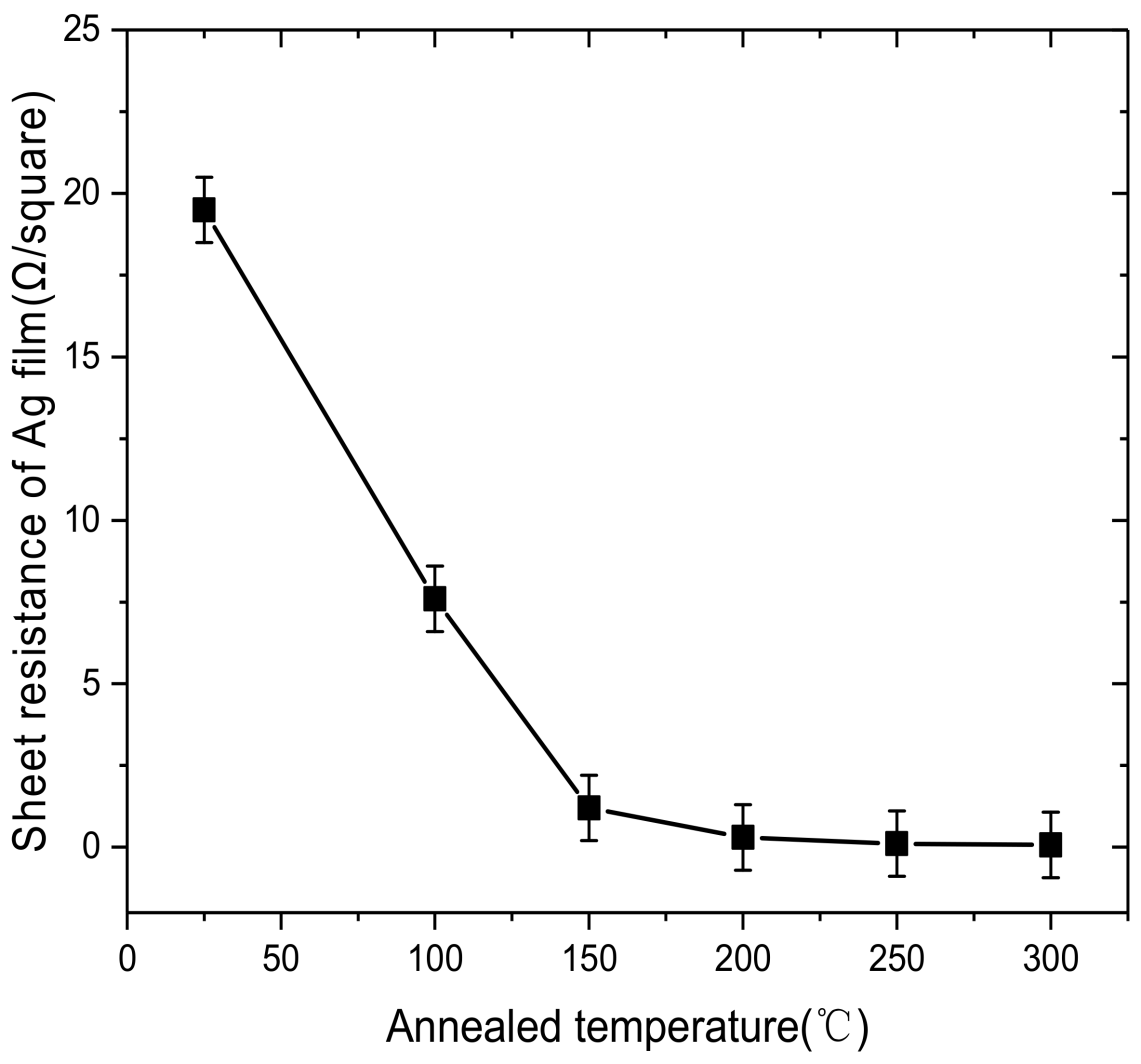
Effect of annealed temperature on sheet resistance of inkjet-printed silver films.

To study the electrical properties of conductive silver ink, it was inkjet-printed into uniform films on UV–Ozone-treated HN-018N photoresist and thermally cured at different temperatures. A DMM-6500 (Keithley, Cleveland, OH, USA) four-point probe sheet resistance tester measured the sheet resistance of silver nanoparticle films at different curing temperatures, deriving the relationship between sheet resistance and curing temperature (Figure 6).

As shown in Figure 6, the sheet resistance of the E2′ silver electrode film inkjet-printed on the photoresist surface decreases significantly with increasing thermal annealed temperature. At room temperature (25 °C), the sheet resistance is ~19.5 Ω/square (ohm per square); it drops sharply to 7.6 Ω/square at 100 °C, and further to ~1.2 Ω/square at 150 °C. With further heating, the conductivity increases and the sheet resistance decreases. However, the reduction rate slows down. The sheet resistance reaches 0.3 Ω/square at 200 °C and 0.11 Ω/square at 250 °C, while no notable change is observed at 300 °C. The sheet resistance curve indicates that conductivity improves with annealed temperature from room temperature to 300 °C, but stabilizes above 150 °C. Below 150 °C, solvents and dispersants in the silver nanoparticle ink evaporate rapidly, and silver nanoparticles interconnect to form a highly conductive silver film. Above 150 °C, solvents and dispersants are nearly fully volatilized, silver nanoparticles are in full contact, and conductivity stabilizes.

### 3.5. Verification of Bistable Characteristics

Two types of BEWD pixels were fabricated successfully—planar electrodes E1/E2 and non-planar electrodes E1/E2′—on a single-layer ITO glass substrate based on photolithography and inkjet printing. Oil remains stable in ON and OFF states without applied voltage.

**Figure 7:**
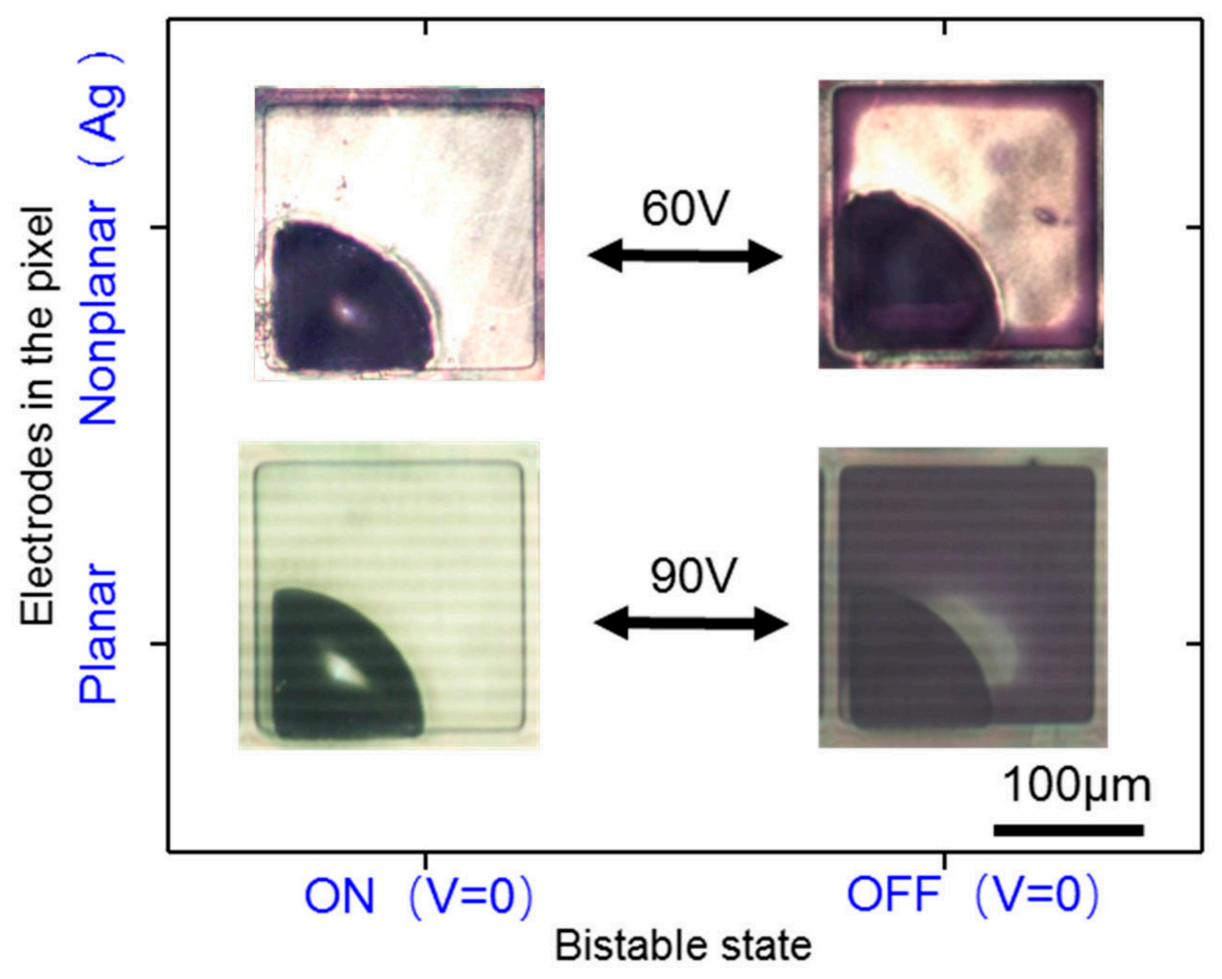
Bi-stability verification of planar (E1 and E2) and non-planar (E1 and E2′) electrodes in BEWD pixels.

Non-planar electrodes outperform planar electrodes with a 30 V lower driving voltage from OFF to ON state, reducing device power consumption and improving reliability. Experimental results are highly consistent with simulation data, verifying the reliability and accuracy of the modeling in the previous work [17]. As shown in Figure 7, the x-axis represents the two bistable ON/OFF states of the pixel, and the y-axis distinguishes planar E1/E2 pixels and non-planar E1/E2′ pixels.

**Figure 8:**
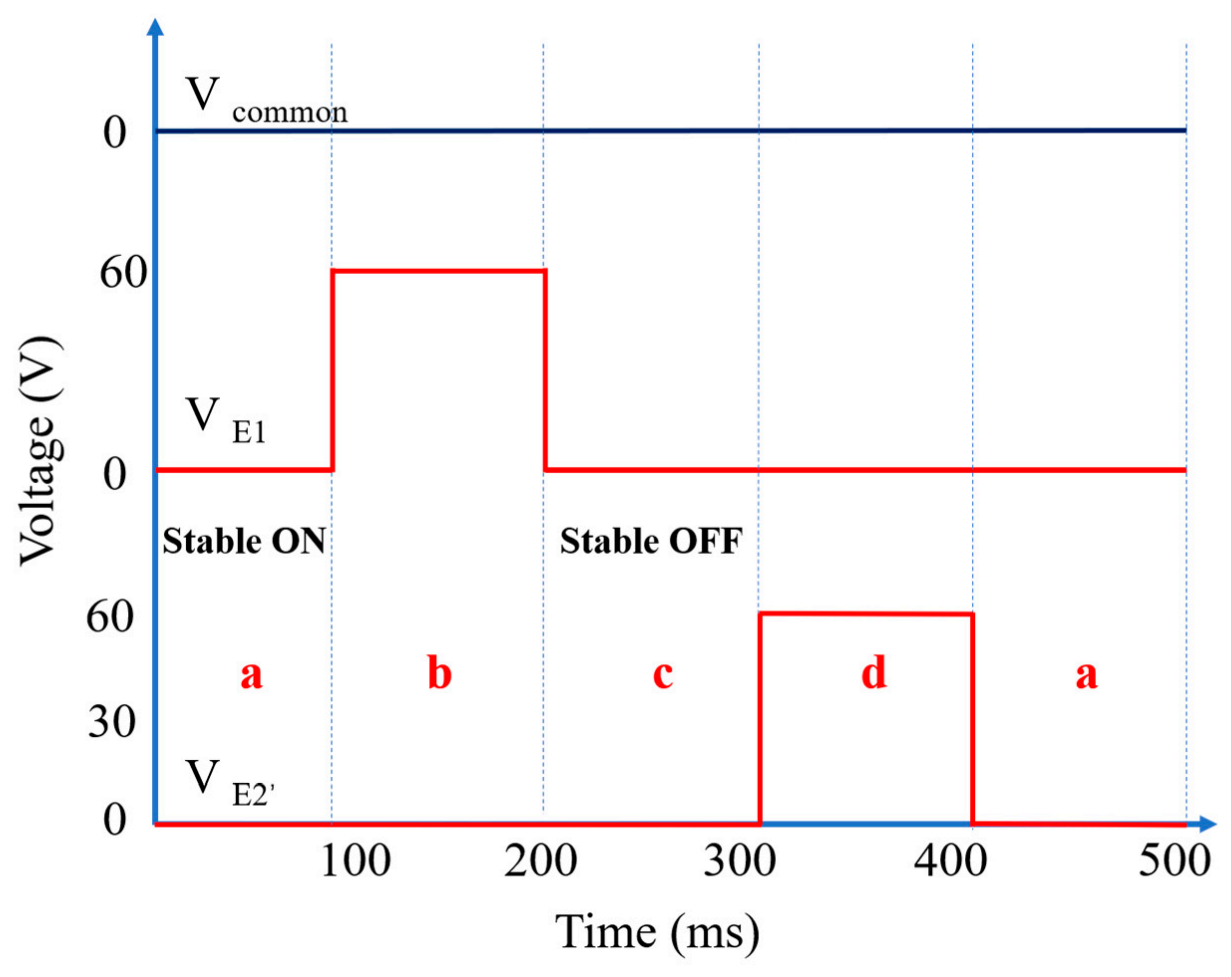
Driving waveform of E1 and E2’ divided into 4 stages (a–d). (**a**) ON state. (**b**) DC 60 V voltage is applied to E1. (**c**) OFF state. (**d**) Applying voltage to planar E2(DC 90V) or non-planar E2′(DC 60V).

The driving waveform is divided into four stages (a to d), as illustrated in Figure 8. Oil was inkjet-printed in both pixel grooves on the bottom electrode E1, maintaining a stable ON state without external voltage (stage a). To verify the OFF state, a DC 60 V voltage is applied to E1, successfully driving oil from the pixel grooves to the pixel step surface for both planar and non-planar pixels (stage b). When voltage is removed, oil spreads freely on the pixel step surface, realizing OFF state (stage c). Reverse switching from OFF back to ON is verified by applying voltage to planar E2 or non-planar E2′ on the pixel step. Planar E2 requires 90 V to drive oil back into grooves, while non-planar E2′ only needs 60 V (stage d).

The planar E2 is located at the bottom of the pixel step, separated from oil by a 12 μm photoresist dielectric layer and 0.8 μm hydrophobic layer. The non-planar E2′ is on the pixel step surface, separated only by a 0.8 μm hydrophobic layer. E2′ generates a larger interfacial tension to drive oil motion at the same applied voltage according to the Young–Lippmann equation.

**Figure 9:**
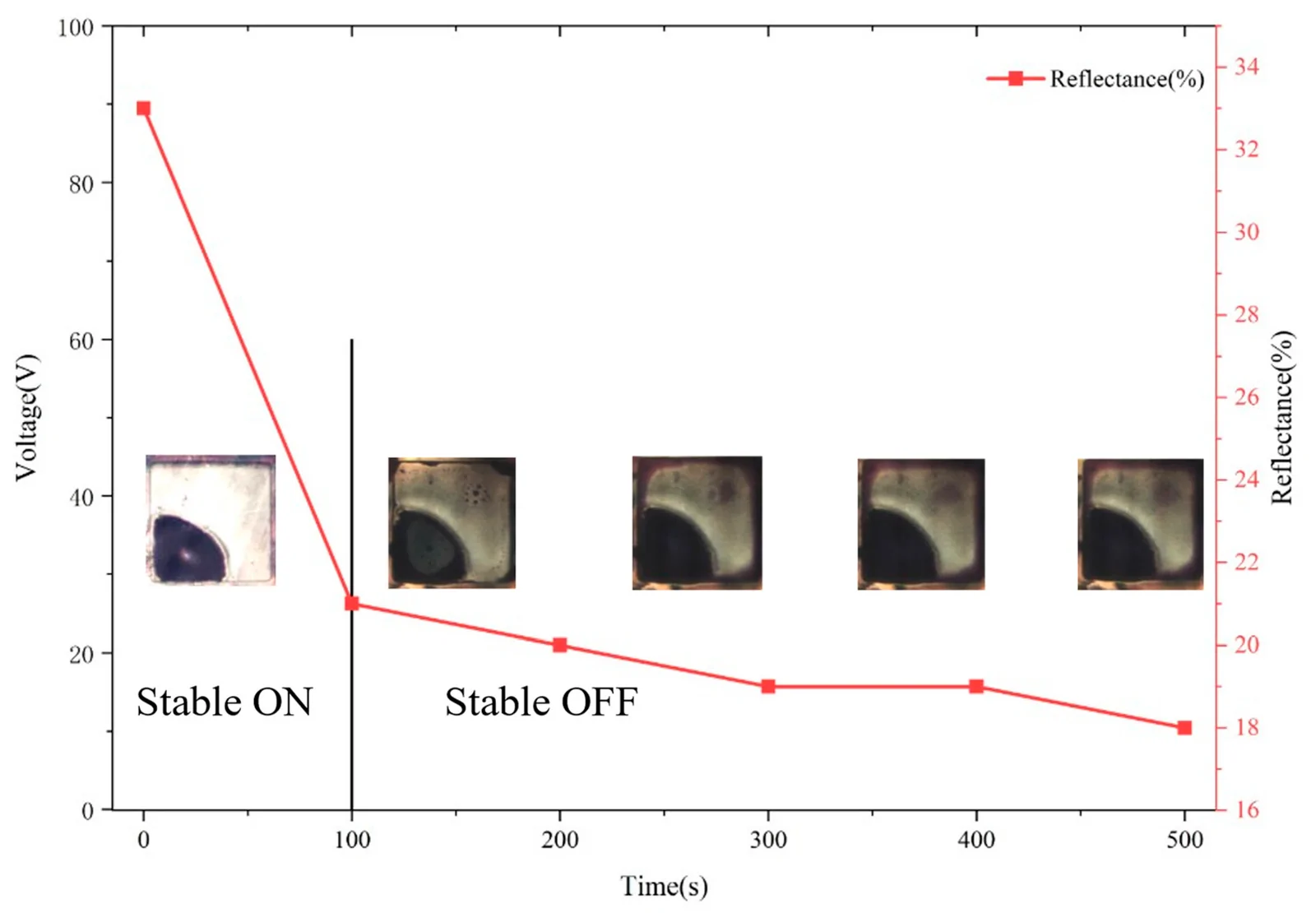
Bi-stability retention of the bistable electrowetting display pixel.

The spectroradiometer (SR-3AR, Topcon, Tokyo, Japan) was set up to measure reflectance to observe bi-stability retention of the BEWD pixel, as shown in Figure 9. The first micrograph shows the ON state of the pixel, and the reflectance is ~33%. Once a 1 s, 60 V pulse was applied to E1, the purple oil in the pixel groove began to rupture and spread on the pixel step, causing the reflectance to drop to ~21%. When the voltage was removed, the oil relaxed onto the pixel step, resulting in an OFF state and the reflectance stayed at roughly 18%. We have been able to verify qualitatively that the pixels remain bistable for several days.

Table 2 summarizes the comparison between this work and previously reported bistable electrowetting display devices. Compared with existing approaches relying on vacuum evaporation, sputtering or laser machining, our strategy combines two-step photolithography, inkjet-printed silver electrodes and inkjet oil filling. Benefiting from the proposed non-planar electrode, a 30 V reduction in OFF-to-ON driving voltage is achieved. The whole fabrication avoids high-vacuum metallization processes and realizes closed pixel cells, demonstrating array-compatible fabrication. Nevertheless, further improvement of device long-term reliability is still required in future study.

## 4. Conclusions

Based on the modeling and simulation data and theoretical support in the previous work [17], this paper successfully fabricates two types of BEWD pixels, planar electrodes E1/E2 and non-planar electrodes E1/E2’, using inkjet printing and photolithography. Both achieve bistable electrowetting display with zero static power consumption, consuming power only during state switching. Non-planar electrodes are superior to planar electrodes with approximately 30 V lower driving voltage from OFF to ON state, benefiting power consumption reduction. Experimental results highly match simulation data, verifying the accuracy of the modeling in the previous work. The fabrication of BEWD devices on a single-layer ITO glass substrate provides insights into the subsequent research and development of flexible, full-color bistable electrowetting display devices.

## Figures and Tables

**Table 1:** Photolithography process parameters for BEWD grooves.

Photolithography Step	Parameters
Photoresist spin coating	Spinner speed: 450 rpm (revolutions per minute), spin time: 65 s
Photoresist pre-baking	Substrate pre-baking temperature: 110 °C, baking time: 15 min
Exposure	Photoresist exposure time: 15 s
Development	Developer solution: 0.4% potassium hydroxide solution, development time: 3 min
Thermal curing	Curing temperature: 150 °C, curing time: 2 h

**Table 2:** Performance comparison between this work and previously reported bistable electrowetting display devices.

Reference	Fabrication Strategy	Driving Voltage	Pixel Feature	Main Limitations
Ref. [14]	Laser-writing, sputtered electrodes	100 V	Planar electrode; open pixel cell	Time-consuming laser processing; poor array compatibility; expensive sputtering facility
Ref. [16]	Photolithography + vacuum-evaporated metal electrodes, manual oil filling	90 V	Open pixel cell	High-vacuum equipment cost; manual oil injection, poor array compatibility; high driving voltage
This work	Two-step photolithography, inkjet-printed Ag electrodes, inkjet oil filling	Reduced by 30 V (OFF to ON)	Non-planar electrode, closed pixel cell, array-compatible, high-resolution potential	Long-term reliability and bistable retention need further optimization

## Data Availability

The original contributions presented in this study are included in the article. Further inquiries can be directed to the corresponding author.
